# Factors Associated with Post-Seasonal Serological Titer and Risk Factors for Infection with the Pandemic A/H1N1 Virus in the French General Population

**DOI:** 10.1371/journal.pone.0060127

**Published:** 2013-04-16

**Authors:** Nathanael Lapidus, Xavier de Lamballerie, Nicolas Salez, Michel Setbon, Rosemary M. Delabre, Pascal Ferrari, Nanikaly Moyen, Marie-Lise Gougeon, Frédéric Vely, Marianne Leruez-Ville, Laurent Andreoletti, Simon Cauchemez, Pierre-Yves Boëlle, Éric Vivier, Laurent Abel, Michaël Schwarzinger, Michèle Legeas, Pierre Le Cann, Antoine Flahault, Fabrice Carrat

**Affiliations:** 1 Institut National de la Santé et de la Recherche Médicale, UMR-S 707, Paris, France; 2 Université Pierre et Marie Curie-Paris 6, UMR-S 707, Paris, France; 3 Unité des Virus Emergents, UMR-D 190, Aix-Marseille université and Institut de Recherche pour le Développement, Marseille, France; 4 Laboratoire de Virologie, Pôle hospitalier de Microbiologie et Maladies Infectieuses, Assistance Publique, Hôpitaux de Marseille, France; 5 Ecole des Hautes Etudes en Sante Publique, Rennes, France; 6 Institut Pasteur, Antiviral Immunity, Biotherapy and Vaccine Unit, Paris, France; 7 Centre d'Immunologie de Marseille-Luminy (CIML), Université de la Méditerranée UM 631, Campus de Luminy, Marseille, France; 8 Institut National de la Santé et de la Recherche Médicale, UMR-S 631, Marseille, France; 9 CNRS, UMR6102, Marseille, France; 10 Assistance Publique, Hôpitaux de Marseille, Hôpital de la Conception, Marseille, France; 11 Université Paris Descartes, Sorbonne Paris Cité, EA 36-20, Paris, France; 12 Laboratoire de Virologie, Hôpital Necker, AP-HP, Paris, France; 13 Unité de Virologie Médicale et Moléculaire, Centre Hospitalier Universitaire, Reims, France; 14 IFR 53/EA-4303 (DAT/PPCIDH), Faculté de Médecine, Reims, France; 15 Medical Research Council Centre for Outbreak Analysis and Modeling, Department of Infectious Disease Epidemiology, Imperial College, London, United Kingdom; 16 Assistance Publique-Hôpitaux de Paris, Hôpital Saint Antoine, Unité de Santé Publique, Paris, France; 17 Laboratoire de Génétique Humaine des Maladies Infectieuses, Institut National de la Santé et de la Recherche Médicale, U 550, Paris, France; 18 Laboratory of Human Genetics of Infectious Diseases, Rockefeller Branch, Rockefeller University, New York, New York, United States of America; 19 Université Paris-Diderot, Sorbonne Paris Cité, UMR 738, Paris, France; 20 Équipe ATIP/AVENIR, Inserm, UMR 738, Paris, France; University of Hong Kong, Hong Kong

## Abstract

The CoPanFlu-France cohort of households was set up in 2009 to study the risk factors for infection by the pandemic influenza virus (H1N1pdm) in the French general population. The authors developed an integrative data-driven approach to identify individual, collective and environmental factors associated with the post-seasonal serological H1N1pdm geometric mean titer, and derived a nested case-control analysis to identify risk factors for infection during the first season. This analysis included 1377 subjects (601 households). The GMT for the general population was 47.1 (95% confidence interval (CI): 45.1, 49.2). According to a multivariable analysis, pandemic vaccination, seasonal vaccination in 2009, recent history of influenza-like illness, asthma, chronic obstructive pulmonary disease, social contacts at school and use of public transports by the local population were associated with a higher GMT, whereas history of smoking was associated with a lower GMT. Additionally, young age at inclusion and risk perception of exposure to the virus at work were identified as possible risk factors, whereas presence of an air humidifier in the living room was a possible protective factor. These findings will be interpreted in light of the longitudinal analyses of this ongoing cohort.

## Introduction

Since the novel influenza A/H1N1 pandemic virus (H1N1pdm) started spreading in April 2009, several studies have identified risk factors for H1N1pdm infection in the community such as young age [1]–[3], ethnicity [2], [4], male gender [4], urban area [5], low pre-epidemic serologic titer [3]–[5], use of public transport [4], household size [6]–[9] and presence of an index case in the household [3], especially if it was a child [10].

The CoPanFlu-France cohort, which has previously been described elsewhere [11], aimed at studying the risk of influenza infection as a complex combination of biological characteristics (including immunity), individual or collective behaviors and environmental context. This integrative approach consists in comprehensively collecting and analyzing epidemiological data on subjects and their environment as well as biological samples [12], [13].

Inclusion of households started in December 2009, at the end of the first H1N1pdm season in metropolitan France. We studied factors associated with the post-pandemic H1N1pdm titer from blood samples collected at inclusion. Previous studies showed that post-pandemic titer was linked to age classes [2], [14]–[20] and to pandemic vaccination status [21]. Relying on the massive amount of data collected at entry in the cohort, we tried to find other independent associations with this titer. In a complementary study, we carried out a nested case-control analysis in these subjects to identify risk factors for probable infection during the first H1N1pdm season.

## Materials and Methods

### Study design

This study relies on 601 households (1450 subjects) included in the study between December 2009 and July 2010, according to a stratified geographical sampling scheme in the French general population. More details on this sampling procedure, the representativeness of the sample and the global study design are available in a previous publication [11]. A total of 575 households (96%) were included after the first pandemic season (September 7 to December 27, 2009 [22]).

During the inclusion visit, nurses collected detailed data from all subjects with questionnaires and blood samples for serological analyses. As 73 of these samples (5.0%) were either too difficult to obtain (young children especially) or of insufficient quality or quantity to be analyzed, the analyses presented here focused on the 1377 subjects for whom haemagglutination inhibition (HI) titer was measured.

### Variables

#### HI assay

The outcome measure was the post-seasonal HI titer, measured from blood samples collected at inclusion. A standard HI technique was adapted to the detection and quantification of antibodies to H1N1pdm. HI assay was conducted in a Bio-Safety Level 3 laboratory using 5.33 haemagglutinating units of non-inactivated antigen [14]. The antigen used was made of a dilution of cell culture supernatant of a H1N1pdm strain (strain OPYFLU-1 isolated from a young patient returning from Mexico in early May 2009) [23]. A final volume of 75 µl was used, including 25 µl of serum dilution, 25 µl of virus suspension, and 25 µl of a 1% RBC suspension in PBS (v/v: 0.33%). The HI titer was determined as the highest dilution providing clear inhibition of haemagglutination in two independent readings [24]. All experiments were conducted using serial dilutions (1/10–1/1280) of heat-inactivated sera, group O human erythrocytes (French Blood Bank). All experiments were performed with same negative and positive controls [25] and with a serum agglutinating activity control. All steps of HI assay were performed on Eppendorf epMotion working stations.

#### Definition of infections (case-control analysis)

Though some authors previously carried out risk factors analyses after defining cases as subjects with HI titer ≥1/40 [2], [26], we chose in our main analysis a higher threshold for our definition as titers between 1/40 and 1/80 were likely to result from a cross-reaction. We therefore defined cases as subjects with HI titer ≥1/80 and all other subjects were considered as controls. In two sensitivity analyses, we additionally defined (i) controls as subjects with HI titer <1/40 and (ii) cases as subjects with HI titer ≥1/80 who reported an influenza-like illness (ILI) during the pandemic season and controls as subjects with HI titer <1/40 and no history of ILI. All pandemic vaccine recipients were excluded from these analyses.

#### Covariates

All covariates used in the analysis are listed elsewhere [11] and detailed in Tables S1–S6 in File S1. The relation with HI titer was studied for 310 covariates, gathered according to 6 main dimensions: 1) sociodemographic characteristics, smoking habits and medical history, 2) vaccination and preventive measures against the virus, 3) indoor housing, 4) attitudes, beliefs and risk perception, 5) nature of meetings with other people and characteristics of contacts and 6) ecological data regarding the surrounding environment. For dimensions 1 to 5, we used data collected from questionnaires completed by the household members, with the help of the visiting nurse. For geographic data, we geocoded the addresses of households and used information on the surrounding demographic and socio-economic context provided by the French Institut national de la statistique et des études économiques (Insee) regarding statistical block groups of about 2000 inhabitants (IRIS) [27].

#### Definitions and coding

Some quantitative covariates were either dichotomized or log-transformed to enhance log-linearity of the studied relation (see supplementary material for details). Age was studied as a quantitative covariate. Subjects reported medical history and vaccinations with the help of their health records. We defined history of ILI as fever ≥37.8°C and cough and/or sore throat without another known cause [28] between September 7, 2009 and the date of inclusion. This covariate was excluded from the case-control analysis, which focused on possible risk factors. Daily frequency of hand washing was reported for the day before inclusion. For covariates describing smoking habits and preventive measures against the virus, characteristics of other members of the household was studied as an individual explanatory covariate (as a mean for quantitative covariates and as a proportion for binary covariates). Covariates regarding attitudes, beliefs and risk perception were collected from all subjects aged over 15 years with a dedicated questionnaire. Subjects were proposed affirmative sentences and were asked for all of them if they totally agreed, partly agreed, partly disagreed or totally disagreed. These answers were dichotomized (agree/disagree).

A contact was defined as someone the subject either spoke with (at least 3 words) or had a physical contact with. All subjects reported meetings with their contacts during a 3-day period ending the day before inclusion. Duration and location of meetings were collected, as well as age of contacts. In order to study meetings as covariates likely to be associated with the HI titer, we summed the individual durations of daily meetings according to their location (home, work, transports and at school) and to the age of contacts respectively. Summed durations of meetings were log-transformed (with an imputed value of 0.01 minute for subjects reporting a null summed duration of meetings). No information was collected on simultaneity of meetings, and the total reported duration of meetings was additional (e.g., a 10-minute meeting with 3 contacts simultaneously accounted for 30 minutes of meeting).

### Statistical methods

All collected covariates likely to be associated with post-seasonal elevated HI titer were studied. Comparison tests between subgroups were Fisher's exact test (for binomial covariates) and Kruskal-Wallis rank sum test (for continuous covariates).

#### Estimation of geometric mean titers (GMTs)

GMTs were estimated for HI assays with the use of regression models for interval-censored data [29], [30] accounting for the within-household correlation. Post-stratification was used to compute representative post-seasonal GMT in the French general population. Calculation and use of sampling weights were detailed elsewhere [11].

We defined the “GMT ratio” (GMTR) as the multiplicative factor between the GMT in exposed versus non-exposed (for a binary covariate) or for each unit increase (for a quantitative covariate).

#### Control for confounding

As age and pandemic vaccination status had an important impact on the serological titer, GMTR was systematically adjusted on these major confounders in all univariable analyses. For analyses regarding environmental characteristics of the bedroom and of the IRIS, correlations were considered at these two levels respectively. Analyses of contacts were adjusted on the proportion of weekend days in the 3-day period.

#### Case-control analysis

Risk factors for infection were studied with the use of alternating logistic regression to model the pairwise odds ratios (ORs) between responses of subjects living in the same room, the same household or the same IRIS [31]. All univariable analyses were adjusted on age and control for confounding was carried out with the same adjustment measures as those used for the GMT analysis.

#### Model selection

The same selection process was used for both analyses. GMTRs and ORs were estimated for all covariates individually. Since a large number of covariates were tested, we adjusted the p-value to control the alpha inflation associated with multiple hypothesis testing and to account for the false discovery rate (FDR) for all covariates [32].

All covariates with an adjusted *P*<0.30 in univariable analyses were included in a multivariable analysis. Thirty datasets were imputed via multiple imputations by chained equations (MICE) [33]. Covariates related to attitudes, beliefs and risk perception for children were sampled from subjects over 15 years in the same household.

The criterion for model selection was the mean residual sum of squares with 10-fold cross-validation – aimed at avoiding overfitting and controlling FDR – run over the 30 imputed datasets and considering only models with *P*<0.05 for all covariates.

Resulting coefficients and standard errors were combined to obtain the reported results [34]. Additional multivariable models were estimated separately stratified by pandemic vaccination status.

Statistical analyses were performed with R version 2.15. We estimated GMTRs with the function “survreg” (package “survival” version 2.36). Multiple imputation was done with the package “mice” and we ran alternating logistic regression with the package “orth”.

## Results

### Descriptive data

Characteristics of the 1377 subjects are given in Tables S1–S6 in File S1. The median age at inclusion was 43.1 years (interquartile range (IQR): 20.7, 59.9 years), 38 children were aged 2 to 5 years and 14 children were aged <2. A total of 561 subjects (40.1%) had at least one history of chronic disease. History of ILI since the beginning of the pandemic wave was reported in 99 subjects (7.5%). For the 3 previous seasons, the proportion of ILI ranged from 7.3% to 19.1%. History of smoking was reported in 544 subjects (39.5%).

The proportion of pandemic vaccine recipients was 12.2%. The median time since pandemic vaccination was 3.2 (IQR: 1.7, 5.2) months. Pandemic vaccine recipients were younger than seasonal vaccine recipients: 46.4 (17.1, 63.0) vs. 63.8 (IQR: 50.4, 72.0) years (*P*<0.0001). Nine hundred and thirteen subjects (66.3%) were living in a house and the median area per inhabitant was 36.7 (IQR: 25.0, 52.5) m^2^.

**Figure 1:**
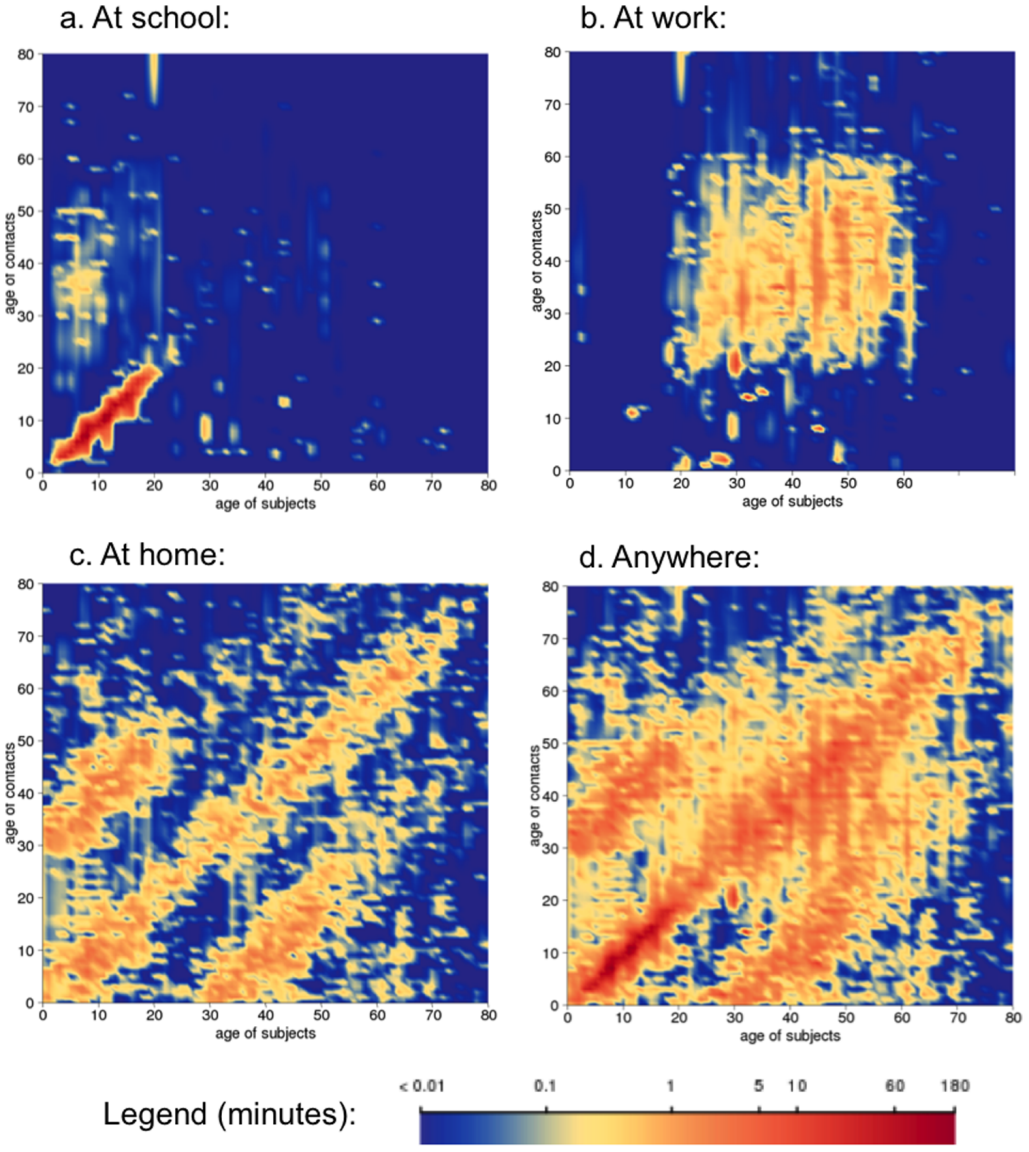
Mean duration of daily meetings (in minutes) of CoPanFlu subjects according to location, age of subjects (±6 months) and age of contacts (±6 months), with 3-year smoothing for both axes.

Detailed data on meetings was collected in 1360 out of the 1377 subjects. The median number of reported daily meetings was 6 (IQR: 3, 10) and the median summed duration was 963 (IQR: 503, 1646) mn/day, with significant differences according to age groups and locations (Figure 1). Subjects aged less than 15 years had a higher daily duration of meetings than older ones: 1847 (IQR: 1147, 2564) mn vs. 848 (IQR: 440, 1378) mn, *P*<0.0001. Children at school reported a large amount of meetings with children of the same age. Working adults aged 20 to 60 years had many meetings with persons of their age. At home, subjects had meetings with people of their age and with persons from the previous or next generation.

### GMT estimates (post-stratified estimates)

Raw measured HI titer was ≥1/20 for 1319 subjects (95.8%), ≥1/40 for 832 subjects (60.4%), ≥1/80 for 259 subjects (18.8%) and ≥1/160 for 50 subjects (3.6%). After post-stratification the estimated proportions were 95.3% for ≥1/20, 59.0% for ≥1/40, 16.1% for ≥1/80 and 2.8% for ≥1/160.

**Figure 2:**
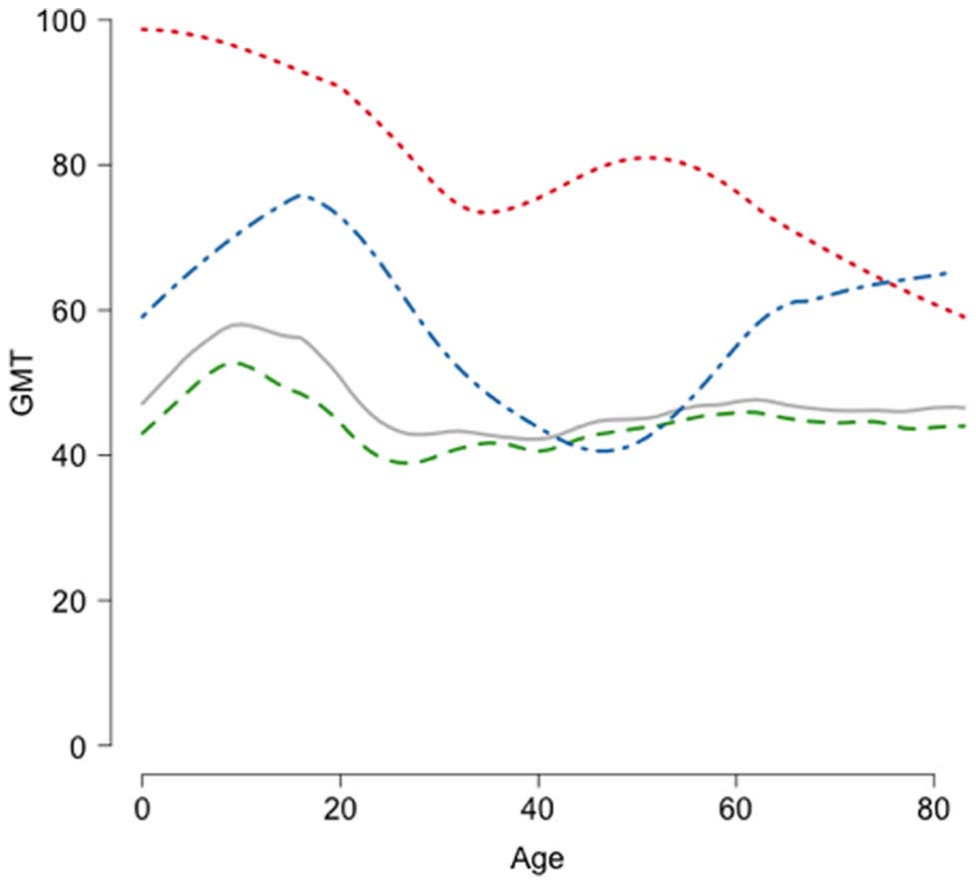
Geometric mean titer (GMT) in relation to age and pandemic vaccination in the general population. Red curve: pandemic vaccine recipients (N = 157); green curve: subjects with no pandemic vaccine and no history of influenza-like illness (ILI) (N = 1,067); blue curve: subjects with no pandemic vaccine and history of ILI (N = 95); gray curve: all subjects (N = 1,377). Smoothed GMTs are estimated for subjects aged between 5 years below and 5 years above the indicated age. GMTs are estimated for each interval based on all subjects in the interval and post-stratified with respect to the general population structure.

The estimated GMT for the general population was 47.1 (95% confidence interval (CI): 45.1, 49.2]) It was higher in pandemic vaccine recipients (80.3 (95% CI: 69.8, 92.5) vs. 44.2 (95% CI: 42.4, 46.0) for unvaccinated subjects with no history of ILI and 58.7 (95% CI: 51.7, 66.6) for unvaccinated subjects with history of ILI) and in subjects aged ess than 5 years (55.2 (95% CI: 49.2, 62.0) vs. 45.7 (95% CI: 43.8, 47.6) for older ones). Figure 2 gives an overview of the estimated post-stratified GMT with respect to the general population structure, in relation to pandemic vaccination status, history of ILI and age of subjects.

### Factors associated with the GMT

All univariable GMTR estimates are listed in Tables S1–S6 in File S1. A total of 40 covariates with adjusted *P*<0.30 were retained in the multivariate analysis.

Selected multivariable models are listed in Table 1. Considering all the subjects (irrespective of the vaccination status), the final model retained (i) pandemic vaccination, 2009 seasonal vaccination, history of ILI for season 2009–2010, asthma, COPD, duration of meetings at school and IRIS proportion of workers using public transports as covariates associated with a higher GMT, and (ii) history of smoking as covariate associated with a lower GMT.

**Table 1:** Multivariable models for geometric mean titer ratio in CoPanFlu-France subjects at inclusion.

*All subjects (N = 1377)*			
Covariate	GMTR	95% CI	P
Pandemic vaccine recipient ^(B)^	1.77	1.56, 2.01	<0.0001
Seasonal vaccine recipient (2009) ^(B)^	1.11	1.01, 1.21	<0.03
History of ILI for season 2009–2010 ^(B)^	1.31	1.15, 1.49	<0.0001
Asthma ^(B)^	1.17	1.01, 1.37	<0.05
Chronic obstructive pulmonary disease ^(B)^	1.28	1.05, 1.56	<0.02
History of smoking ^(B)^	0.93	0.88, 0.99	<0.03
Duration of meetings at school ^(L)^	1.03	1.01, 1.04	<0.01
Proportion of workers using public transports to go to work ^(Q)^	1.45	1.00, 2.10	<0.05

*Subjects without pandemic vaccination (N = 1207)*			
Covariate	GMTR	95% CI	P
Seasonal vaccine recipient (2009) ^(B)^	1.30	1.13, 1.48	<0.001
History of ILI for season 2009–2010 ^(B)^	1.11	1.03, 1.20	<0.01
Chronic obstructive pulmonary disease ^(B)^	1.23	1.02, 1.48	<0.03
History of smoking ^(B)^	0.92	0.86, 0.98	<0.02
Duration of meetings at school ^(L)^	1.03	1.01, 1.04	<0.001
Proportion of workers using public transports to go to work^(Q)^	1.46	1.01, 2.11	<0.05

*Pandemic vaccine recipients (N = 168)*			
Covariate	GMTR	95% CI	P
Age at inclusion (per 10 years) ^(Q)^	0.91	0.86, 0.96	<0.001
History of ILI for season 2009–2010 ^(B)^	1.62	1.39, 1.90	<0.0001
Months since pandemic vaccination ^(B)^	0.84	0.79, 0.90	<0.0001

(B): binary covariates; (Q): quantitative covariates; (L): log-transformed quantitative covariates; GMTR: geometric mean titer ratio; CI: confidence interval.

Considering the 1,207 subjects without pandemic vaccination, “asthma” was the only covariate that did not remain in the final model. Considering the 171 pandemic vaccine recipients, history of ILI remained in the model, while older age at inclusion and time since pandemic vaccination were associated with a lower GMT.

### Case-control analysis

The 1,207 unvaccinated subjects were included in this analysis, 171 as cases and 1,036 as controls. The proportions of subjects with a history of ILI were 18.0% in cases and 6.5% in controls (*P*<0.0001). The final multivariable model retained (i) COPD, asthma, duration of meetings at school, proportion of workers using public transports and belief that not going to work protects against H1N1pdm as factors associated with a higher risk of probable infection, and (ii) older age and having an air humidifier in the living room as factors associated with a lower risk (see Table 2 for details). Though we estimated pairwise odds ratios between responses of subjects living in the same room, the same household or the same IRIS, only the household level was kept in the final model as the other ones were not significant, OR  = 3.31 (95% CI: 1.82, 6.02). Multivariable models for the sensitivity analyses retained subsets of these factors with no additional factors (Table S7 and S8 in File S2).

**Table 2:** Multivariable models for the case-control analysis of risk factors for probable infection in CoPanFlu-France unvaccinated subjects.

*Subjects without pandemic vaccination, 171 cases with HI titer ≥1/80, 1,036 controls with HI titer <1/80*			
Covariate	OR	95% CI	P
Age at inclusion (per 10 years) ^(Q)^	0.87	0.77, 0.98	<0.02
Chronic obstructive pulmonary disease ^(B)^	2.89	1.41, 5.92	<0.01
Asthma ^(B)^	2.41	1.32, 4.42	<0.01
Duration of meetings at school ^(L)^	1.11	1.03, 1.19	<0.01
Air humidifier in the living room ^(B)^	0.64	0.41, 0.99	<0.05
Believes that not going to work protects against H1N1pdm ^(B)^	1.61	1.02, 2.53	<0.05
Proportion of workers using public transports to go to work ^(Q)^	11.2	2.08, 60.0	<0.01
*Pairwise odds ratios between cases living in the same household*	*3.31*	*1.82, 6.02*	*<0.0001*

(B): binary covariates; (Q): quantitative covariates; (L): log-transformed quantitative covariates; OR: odds ratio; CI: confidence interval.

## Discussion

### Covariates associated with HI titer

Post-pandemic elevated HI titer can be explained by a pre-pandemic elevated titer, a recent increase in titer due to an infection by the pandemic virus or to another antigenic stimulation (e.g., pandemic vaccination), or by any combination of these different factors. We review our findings in light of other studies on the same topic.

The global multivariate model including pandemic vaccination gave information on the association of this factor with the GMT. Adjustment on this factor in the same model allowed us to study factors that may have an impact on GMT increase after either vaccination or infection, whereas stratified analyses according to this vaccination intended to focus more specifically on factors associated with other causes of elevated GMT.

We found a lower anti-H1N1pdm GMT in older subjects in the univariable analysis and in the multivariable model run among pandemic vaccine recipients. This covariate did not remain in the other multivariate models mainly because of the adjustment on duration of contacts at school (age was significantly associated with the GMT in all models when we excluded this covariate). Older age was also associated with a lower risk of probable infection in the case-control analysis.

These results are consistent with other cross-sectional post-pandemic studies worldwide, including modeling [35] and serological [14] studies in France, which reported a much higher infection rate in children and young adults [16], [19], [20], [36]–[44].

**Figure 3:**
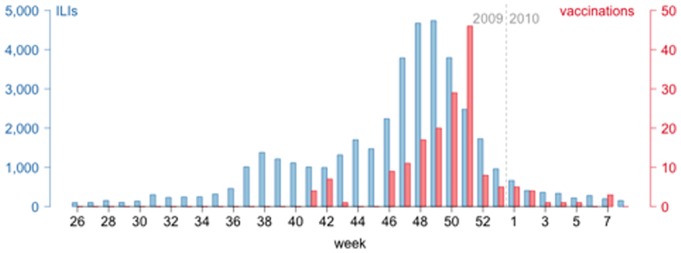
Weekly incidence of influenza-like illnesses (ILIs) in France (French General Practitioner Sentinel network [**22**]) and weekly pandemic vaccinations in CoPanFlu subjects, weeks 2009–26 to 2010–08. Blue bars (left scale): national weekly incidence of ILIs; red bars (right scale): number of weekly pandemic vaccinations in CoPanFlu subjects.

As expected, a reported history of ILI was associated with an elevated GMT, which indicates that some of these ILIs were probably caused by H1N1pdm infection. Though this factor lacks sensitivity and specificity to be considered as a good correlate of infection, its coefficient in selected multivariable models gives more information on the relative role of infections among all causes leading to a GMT increase. Its association with the GMT in vaccinated subjects indicates that the GMT was also caused by H1N1pdm infections. Indeed, as most vaccinations occurred at the end of the pandemic course (Figure 3), we could not distinguish whether the increased GMT in vaccine recipients was caused by vaccination itself or by previous infection.

Asthma and COPD were associated with a higher GMT and possible risk factors in the case-control analysis. Asthmatics may have increased susceptibility for H1N1pdm infection [45], possibly because of alterations in the airway architecture [46], [47] and impairment of innate immunity [47]. Another hypothesis to explain a higher GMT in subjects with such medical conditions, regardless of their susceptibility to infection, would be a more severe illness [48] involving a greater immune response [49].

We found that smoking history was associated with a lower GMT. Although several studies already found an association between cigarette smoking and risk to contract influenza infection [50]–[52], smokers have a well-known diminished serological response to influenza infection or vaccination [52], the immunosuppressive mechanism is still unclear [53]–[56].

Seasonal vaccination for any season since 2006–2007 was associated with an increase in the GMT, maybe because of a cross-reactive immune response with seasonal vaccination H1N1 strains [57]though studies investigating this association were all inconclusive [3], [58]. Another hypothesis would consider that elevated post-seasonal titer might be a consequence of an increased risk of pandemic infection in seasonal vaccine recipients [59], though conflicting results were reported about this association [60]–[64].

In covariates related to the environmental characteristics of the housing, only the association between presence of an air humidifier in the living room and lower risk remained in the case-control multivariable model, which may be consistent with the possible impact of relative humidity on influenza aerosol transmission [65], [66].

The multivariable analysis retained no covariate related to attitudes, beliefs and risk perception, except the belief that not going to work may protect against H1N1pdm infection, associated with a higher risk in the case-control analysis. We have no clear interpretation for this finding, except that this covariate may be a correlate of more general characteristics of risk perception, which affect the transmission patterns of pandemic influenza.

Increasing GMT and a higher risk of probable infection associated with duration of meetings at school were not surprising since schools are identified as places with high meeting rates between influenza susceptible subjects [67]. Interestingly, we did not find a significant association of GMT with daily duration of meetings with children younger than 10 years old regardless of location, suggesting that school favors transmissions by a particular pattern of contacts or environmental characteristics [67], [68].

The multivariable analysis retained no covariate related to the characteristics of the surrounding area, except the proportion of workers using public transportation to go to work, which also appeared as a possible risk factor.

The important pairwise OR we found in the case-control analysis for subjects living in the same household suggests a common environmental exposure or susceptibility for these subjects who often belong to the same family, or more probably an elevated intra-household secondary attack rate (estimated 4 to 37% in previous household studies [10]).

### Limitations

Though households were sampled in the general population, some households refused to participate, which may induce a selection bias. However, comparisons with French population census data suggest that this bias was controlled [11], and post-stratification of the GMT by age and vaccination status with respect to the French population structure did not modify the results significantly. We did not post-stratify our estimations of the GMTR, as the choice of the auxiliary covariates used to adjust the sampling weights could have induced important changes in the standard error of our estimates leading to spurious associations [69].

The timeline of inclusion may have induced recall or reporting biases. The cohort was designed to include households before the 2009 pandemic season and to follow-up subjects during the influenza season. As inclusions were delayed, data regarding ILIs were collected retrospectively and recall bias may be important in subjects with late inclusion. Moreover, we found a decreasing GMT according to time since vaccination in pandemic vaccine recipients, and we cannot exclude an antibody loss in the months following an infection, although we did not find any association between GMT and date of inclusion in unvaccinated subjects. Such limitations may have biased the association between GMT and other covariates.

In the case-control analysis, cases were defined serologically, yet we know that an elevated titer can sometimes be explained by cross-reactions, especially in the elderly [16], and that infected subjects can show a low titer a few months after infection [70]. This lack of specificity and sensitivity to identify infections must be considered in light of the sensitivity analyses results, which often showed similar results with different case definitions.

Another limitation may be linked to the amount of data collected. Though we controlled this FDR with the use of specific procedures, multiple testing of hundreds of covariates results in an important risk of finding spurious associations, due to the alpha inflation phenomenon.

Because of these limitations, our analysis must be understood as a hypothesis generating study aimed at identifying the possible role of many factors that would probably not have been studied otherwise. Further studies would be necessary to confirm the impact of these factors and their implications for the control of influenza.

## Conclusion

We used a data-driven framework to carry out an exploratory analysis of potential relevant risk factors for infection. This hypothesis generating tool relying on an integrated approach allowed us to highlight the possible impact of previously unknown factors from several dimensions usually studied separately, such as presence of an air humidifier (indoor environment), duration of meetings at school (social contacts), characteristics of the local population or risk perception. Additional data is being collected and analyzed in this ongoing cohort. The longitudinal analysis of these households will permit integrative analyses of complex phenomena such as individual, collective and environmental risk factors for infection, routes of transmission, or determinants of the immune response to infection or vaccination.

## Supporting Information

File S1 **Tables S1–S6.** Description and univariable analyses for all covariates.
(DOC)

File S2 **Tables S7 and S8.** Multivariable models for the case-control analysis of risk factors for probable infection in CoPanFlu-France unvaccinated subjects: sensitivity analysis.
(DOC)
