# Supplementary material for: Factors Associated with Post-Seasonal Serological Titer and Risk Factors for Infection with the Pandemic A/H1N1 Virus in the French General Population
Source: PLoS One. 2013 Apr 16;8(4):e60127. doi: 10.1371/journal.pone.0060127 (PMC3629047; doi:10.1371/journal.pone.0060127)
Supplement: File S1 — Tables S1–S6. Description and univariable analyses for all covariates. (DOC) [file pone.0060127.s001.doc]

**Factors associated with post-seasonal serological titer and risk factors for infection with the pandemic A/H1N1 virus in the French general population**

Supplementary tables S1–S6: description and univariable analyses for all covariates

- Table S1: Socio-demographic characteristics, smoking habits and medical history
- Table S2: Preventive measures
- Table S3: Environmental characteristics of the housing
- Table S4: Attitudes, beliefs and risk perception
- Table S5: Nature and daily duration of meetings
- Table S6: Geographic area

All covariates are binary except ^(Q)^ quantitative covariates and ^(L)^ log-transformed quantitative covariates. Covariates regarding “other subjects in the household” are defined for households with at least two subjects, whereas covariates regarding “all subjects in the household” are defined for all subjects.

Quantitative covariates are described as median [interquartile range (IQR)] and binary covariates as N (percentage). Geometric mean titer ratios (GMTR) are given with their 95% confidence interval (CI).

| **Table S1. Socio-demographic characteristics, smoking habits and medical history** | | | | |  |
| --- | --- | --- | --- | --- | --- |
| ***Socio-demographic characteristics*** |  |  |  |  |  |
| **Covariate** | **missing values (N)** | **median ([IQR] or N (proportion)** | **GMTR (95% CI)** | **raw P** | **adjusted P** |
| Sex = male | 0 | 649 (47.1%) | 0.9 (0.85, 0.95) | < 0.001 | < 0.02 |
| Age at inclusion (years) (Q) | 0 | 43.1 [20.7, 59.9] | 1 (1, 1) | < 0.01 | 0.05 |
| Has a professional activity | 0 | 641 (46.6%) | 0.94 (0.89, 1) | 0.06 | 0.32 |
| Professional activity: |  |  |  |  |  |
| - Primarily in enclosed space | 0 | 456 (33.1%) | 0.98 (0.92, 1.05) | 0.64 | 0.66 |
| - Primarily outdoors | 0 | 115 (8.4%) | 0.93 (0.84, 1.03) | 0.19 | 0.48 |
| - Involves contact with children | 0 | 196 (14.2%) | 0.97 (0.89, 1.06) | 0.50 | 0.59 |
| - Involves contact with ill people | 0 | 137 (9.9%) | 0.99 (0.89, 1.1) | 0.86 | 0.70 |
| Household annual income > 36 k€ | 243 | 721 (80.9%) | 0.96 (0.87, 1.05) | 0.38 | 0.56 |
|  |  |  |  |  |  |
| **Medical history** |  |  |  |  |  |
| **Covariate** | **missing values (N)** | **median ([IQR] or N (proportion)** | **GMTR (95% CI)** | **raw P** | **adjusted P** |
| History of ILI: |  |  |  |  |  |
| - Season 2009-2010 | 56 | 99 (7.5%) | 1.33 (1.16, 1.52) | < 0.0001 | < 0.01 |
| - Season 2008-2009 | 0 | 215 (15.6%) | 1.06 (0.98, 1.16) | 0.16 | 0.45 |
| - Season 2007-2008 | 0 | 138 (10%) | 0.97 (0.88, 1.07) | 0.55 | 0.61 |
| - Season 2006-2007 | 0 | 121 (8.8%) | 1.05 (0.94, 1.17) | 0.41 | 0.56 |
|  |  |  |  |  |  |
| Self-rated good health in the last 12 months | 19 | 889 (65.5%) | 1.11 (1.04, 1.19) | < 0.01 | < 0.04 |

| **Medical history (continued)** |  |  |  |  |  |
| --- | --- | --- | --- | --- | --- |
| **Covariate** | **missing values (N)** | **median ([IQR] or N (proportion)** | **GMTR (95% CI)** | **raw P** | **adjusted P** |
| Past or ongoing chronic condition: |  |  |  |  |  |
| - Any | 0 | 561 (40.7%) | 1.04 (0.97, 1.11) | 0.24 | 0.50 |
| - Diabetes | 0 | 67 (4.9%) | 1.14 (1.01, 1.3) | < 0.05 | 0.27 |
| - History of asthma | 0 | 103 (7.5%) | 1.07 (0.94, 1.22) | 0.28 | 0.51 |
| - Ongoing asthma | 0 | 62 (4.5%) | 1.19 (1.00, 1.40) | < 0.05 | 0.28 |
| - Chronic obstructive pulmonary disease | 0 | 56 (4.1%) | 1.24 (1.06, 1.44) | < 0.01 | 0.08 |
| - Hypertension | 0 | 184 (13.4%) | 1.02 (0.93, 1.12) | 0.68 | 0.66 |
| - HIV infection | 0 | 6 (0.4%) | 0.99 (0.67, 1.45) | 0.95 | 0.73 |
| - Chronic renal disease | 0 | 14 (1%) | 0.97 (0.74, 1.28) | 0.84 | 0.70 |
| - Chronic liver disease | 0 | 5 (0.4%) | 0.8 (0.67, 0.97) | < 0.03 | 0.17 |
| - Systemic disease | 0 | 35 (2.5%) | 0.91 (0.77, 1.07) | 0.27 | 0.50 |
| - Chronic respiratory insufficiency | 0 | 29 (2.1%) | 1.11 (0.88, 1.41) | 0.38 | 0.56 |
| - Cardiac insufficiency | 0 | 42 (3.1%) | 1.11 (0.94, 1.31) | 0.21 | 0.48 |
| - Cardiac arrhythmia | 0 | 73 (5.3%) | 1.02 (0.88, 1.17) | 0.82 | 0.70 |
| - Valvular heart disease | 0 | 20 (1.5%) | 0.87 (0.71, 1.07) | 0.18 | 0.48 |
| - Congenital heart disease | 0 | 4 (0.3%) | 1.22 (0.89, 1.67) | 0.22 | 0.49 |
| - Coronary insufficiency | 0 | 48 (3.5%) | 0.9 (0.76, 1.07) | 0.22 | 0.49 |
| - History of myocardial infarction | 0 | 18 (1.3%) | 0.91 (0.7, 1.2) | 0.51 | 0.60 |
| - History of stroke | 0 | 18 (1.3%) | 0.77 (0.63, 0.93) | < 0.01 | 0.08 |
| - Cancer | 0 | 57 (4.1%) | 1.04 (0.9, 1.21) | 0.56 | 0.62 |
| - Breast cancer | 0 | 14 (1%) | 1.03 (0.79, 1.36) | 0.81 | 0.70 |
| - Prostate cancer | 0 | 12 (0.9%) | 0.94 (0.69, 1.28) | 0.69 | 0.67 |
| - Chemotherapy | 0 | 18 (1.3%) | 1.18 (0.93, 1.48) | 0.17 | 0.47 |
| - Radiotherapy | 0 | 27 (2%) | 1.03 (0.87, 1.22) | 0.71 | 0.68 |
| - Chronic neuromuscular disorder | 0 | 33 (2.4%) | 1.07 (0.89, 1.28) | 0.47 | 0.59 |
| - Hypothyroidism | 0 | 5 (0.4%) | 1.11 (0.82, 1.5) | 0.51 | 0.60 |
| - Any dysthyroidism | 0 | 12 (0.9%) | 1.16 (0.9, 1.51) | 0.26 | 0.50 |
| - Multiple sclerosis | 0 | 8 (0.6%) | 0.9 (0.63, 1.3) | 0.59 | 0.63 |
| - Allergy: |  |  |  |  |  |
| - - Any | 0 | 14 (1%) | 1.21 (0.88, 1.66) | 0.24 | 0.50 |
| - - Pollen | 0 | 6 (0.4%) | 1.31 (0.71, 2.4) | 0.39 | 0.56 |
| - - Dust mite | 0 | 4 (0.3%) | 1.1 (0.77, 1.56) | 0.59 | 0.64 |
| - - Egg proteins | 0 | 10 (0.7%) | 0.92 (0.61, 1.37) | 0.68 | 0.66 |
| - Arthosis | 0 | 8 (0.6%) | 1.56 (0.85, 2.88) | 0.15 | 0.45 |
| - Any respiratory disease | 0 | 154 (11.2%) | 1.09 (0.98, 1.21) | 0.10 | 0.41 |
| - Hypercholesterolemia | 0 | 5 (0.4%) | 0.96 (0.58, 1.59) | 0.87 | 0.70 |
| - Depression | 0 | 10 (0.7%) | 0.87 (0.62, 1.22) | 0.41 | 0.56 |
| - Epilepsy | 0 | 10 (0.7%) | 1.35 (0.94, 1.93) | 0.11 | 0.41 |
| - Herniated disc | 0 | 7 (0.5%) | 0.78 (0.56, 1.08) | 0.14 | 0.44 |
| - Migraine, headaches | 0 | 4 (0.3%) | 0.79 (0.6, 1.04) | 0.09 | 0.40 |
| - Tuberculosis | 0 | 7 (0.5%) | 0.89 (0.65, 1.22) | 0.47 | 0.59 |
| - Pneumonia | 0 | 5 (0.4%) | 0.77 (0.36, 1.63) | 0.49 | 0.59 |
| - Coronary bypass | 0 | 3 (0.2%) | 1.27 (0.65, 2.49) | 0.49 | 0.59 |
| - Immunodepression | 0 | 105 (7.6%) | 1.05 (0.94, 1.17) | 0.37 | 0.56 |
| - Any cardiovascular disease | 0 | 246 (17.9%) | 0.98 (0.9, 1.07) | 0.63 | 0.66 |
|  |  |  |  |  |  |
|  |  |  |  |  |  |
| ***Smoking habits*** |  |  |  |  |  |
| **Covariate** | **missing values (N)** | **median ([IQR] or N (proportion)** | **GMTR (95% CI)** | **raw P** | **adjusted P** |
| History of smoking | 0 | 544 (39.5%) | 0.9 (0.85, 0.96) | < 0.001 | < 0.02 |
| Current smoker | 0 | 267 (19.4%) | 0.88 (0.82, 0.95) | < 0.001 | < 0.02 |
| Smoke inside home | 0 | 149 (10.8%) | 0.93 (0.85, 1.02) | 0.13 | 0.44 |
| Smoke inside home even when not alone | 0 | 101 (7.3%) | 0.94 (0.85, 1.05) | 0.29 | 0.51 |
| Smoke inside home even without open window | 0 | 100 (7.3%) | 0.93 (0.84, 1.03) | 0.18 | 0.48 |
| Smoke inside car | 0 | 122 (8.9%) | 0.84 (0.76, 0.93) | < 0.001 | < 0.02 |
| Smoke inside car even when not alone | 0 | 58 (4.2%) | 0.84 (0.72, 0.98) | < 0.03 | 0.18 |
| Smoke inside car even without open window | 0 | 19 (1.4%) | 0.85 (0.68, 1.07) | 0.16 | 0.45 |
|  |  |  |  |  |  |
|  |  |  |  |  |  |
|  |  |  |  |  |  |
| ***Household characteristics*** |  |  |  |  |  |
| **Covariate** | **missing values (N)** | **median ([IQR] or N (proportion)** | **GMTR (95% CI)** | **raw P** | **adjusted P** |
| History of ILI in other subjects of the household: |  |  |  |  |  |
| - Season 2009-2010 (Q) | 189 | 0 [0, 0] | 1.05 (0.91, 1.22) | 0.48 | 0.59 |
| - Season 2008-2009 (Q) | 134 | 0 [0, 0.25] | 0.98 (0.87, 1.11) | 0.74 | 0.69 |
| - Season 2007-2008 (Q) | 134 | 0 [0, 0] | 1.01 (0.87, 1.18) | 0.91 | 0.72 |
| - Season 2006-2007 (Q) | 134 | 0 [0, 0] | 1.05 (0.88, 1.24) | 0.60 | 0.64 |
|  |  |  |  |  |  |
| History of ILI in all subjects of the householdd: |  |  |  |  |  |
| - Season 2009-2010 (Q) | 56 | 0 [0, 0] | 1.33 (1.11, 1.58) | < 0.01 | < 0.03 |
| - Season 2008-2009 (Q) | 0 | 0 [0, 0] | 1 (0.86, 1.16) | 0.99 | 0.74 |
| - Season 2007-2008 (Q) | 0 | 0 [0, 0] | 1.09 (0.92, 1.28) | 0.32 | 0.52 |
| - Season 2006-2007 (Q) | 56 | 0 [0, 0] | 1.33 (1.11, 1.58) | < 0.01 | < 0.03 |
|  |  |  |  |  |  |
| Smoking habits in other subjects of the household: |  |  |  |  |  |
| - History of smoking (Q) | 134 | 0.33 [0, 0.67] | 1.04 (0.95, 1.13) | 0.42 | 0.57 |
| - Current smoker (Q) | 134 | 0 [0, 0.33] | 0.99 (0.89, 1.11) | 0.92 | 0.72 |
| - Smoke inside home (Q) | 134 | 0 [0, 0] | 1.09 (0.95, 1.25) | 0.24 | 0.50 |
|  |  |  |  |  |  |
| Smoking habits in all subjects of the household: |  |  |  |  |  |
| - History of smoking (Q) | 0 | 0.33 [0, 0.5] | 0.94 (0.85, 1.04) | 0.22 | 0.49 |
| - Current smoker (Q) | 0 | 0 [0, 0.33] | 0.91 (0.8, 1.03) | 0.12 | 0.43 |
| - Smoke inside home (Q) | 0 | 0 [0, 0] | 1.04 (0.92, 1.18) | 0.55 | 0.61 |
|  |  |  |  |  |  |

| **Table S2. Preventive measures** | | | | |  |
| --- | --- | --- | --- | --- | --- |
| ***Vaccination*** |  |  |  |  |  |
| **Covariate** | **missing values (N)** | **median ([IQR] or N (proportion)** | **GMTR (95% CI)** | **raw P** | **adjusted P** |
| Pandemic vaccination (2009) | 2 | 168 (12.2%) | 1.71 (1.51, 1.94) | < 0.0001 | < 0.0001 |
| Seasonal vaccination |  |  |  |  |  |
| - Season 2009-2010 | 2 | 304 (22.1%) | 1.19 (1.1, 1.3) | < 0.0001 | < 0.01 |
| - Season 2008-2009 | 5 | 287 (20.9%) | 1.14 (1.04, 1.24) | < 0.01 | 0.07 |
| - Season 2007-2008 | 8 | 275 (20.1%) | 1.11 (1.02, 1.22) | < 0.02 | 0.16 |
| - Season 2006-2007 | 11 | 268 (19.6%) | 1.12 (1.02, 1.23) | < 0.02 | 0.15 |
| - Any season from 2006-2007 to 2008-2009 | 10 | 330 (24.1%) | 1.14 (1.04, 1.24) | < 0.01 | < 0.05 |
| Anti-pneumococcal vaccination | 46 | 93 (7%) | 1.07 (0.93, 1.24) | 0.34 | 0.54 |
|  |  |  |  |  |  |
|  |  |  |  |  |  |
| ***Hygiene measures*** |  |  |  |  |  |
| **Covariate** | **missing values (N)** | **median ([IQR] or N (proportion)** | **GMTR (95% CI)** | **raw P** | **adjusted P** |
| Using handkerchief when wiping nose | 0 | 252 (18.3%) | 0.91 (0.83, 0.99) | < 0.04 | 0.24 |
| Using (paper) tissue when wiping nose | 0 | 1196 (86.9%) | 1.05 (0.95, 1.15) | 0.35 | 0.54 |
| Using antiviral (paper) tissue when wiping nose | 0 | 7 (0.5%) | 0.91 (0.69, 1.19) | 0.48 | 0.59 |
| Always covers mouth while coughing or sneezing | 17 | 696 (51.2%) | 1.04 (0.97, 1.11) | 0.25 | 0.50 |
| Daily frequency of hand washing  (with soap or hand sanitizer) ≥ 3 (B) (Q) | 36 | 5 [4, 10] | 1 (0.99, 1) | 0.51 | 0.60 |
| Always washes hands after coughing or sneezing | 21 | 113 (8.3%) | 1.02 (0.93, 1.11) | 0.75 | 0.69 |
|  |  |  |  |  |  |
|  |  |  |  |  |  |
| ***Household characteristics*** |  |  |  |  |  |
| **Covariate** | **missing values (N)** | **median ([IQR] or N (proportion)** | **GMTR (95% CI)** | **raw P** | **adjusted P** |
| Preventive measures in other subjects of the household: |  |  |  |  |  |
| - Seasonal vaccination 2009-2010 (Q) | 137 | 0 [0, 0.25] | 1.09 (0.99, 1.21) | 0.08 | 0.40 |
| - Any seasonal vaccination from 2006-2007 to 2008-2009 (Q) | 138 | 0 [0, 0.33] | 1.06 (0.96, 1.18) | 0.25 | 0.50 |
| - Using handkerchief when wiping nose (Q) | 134 | 0 [0, 0.17] | 1.07 (0.97, 1.18) | 0.19 | 0.48 |
| - Using (paper) tissue when wiping nose (Q) | 134 | 1 [1, 1] | 0.96 (0.86, 1.08) | 0.50 | 0.59 |
| - Using antiviral (paper) tissue when wiping nose (Q) | 134 | 0 [0, 0] | 1.19 (0.73, 1.91) | 0.49 | 0.59 |
| - Always covers mouth while coughing or sneezing (Q) | 143 | 0.5 [0, 1] | 1.06 (0.97, 1.16) | 0.18 | 0.48 |
| - Daily frequency of hand washing  (with soap or hand sanitizer) ≥ 3 (B) (Q) | 150 | 6 [4, 8.67] | 1 (0.99, 1.01) | 0.75 | 0.69 |
| - Always washes hands after coughing or sneezing (Q) | 142 | 0 [0, 0] | 0.97 (0.83, 1.12) | 0.67 | 0.66 |
|  |  |  |  |  |  |
|  |  |  |  |  |  |

| ***Household characteristics (continued)*** | | |  |  |  |
| --- | --- | --- | --- | --- | --- |
| **Covariate** | **missing values (N)** | **median ([IQR] or N (proportion)** | **GMTR (95% CI)** | **raw P** | **adjusted P** |
| Preventive measures in all subjects of the household: |  |  |  |  |  |
| - Seasonal vaccination 2009-2010 (Q) | 0 | 0 [0, 0.33] | 1.21 (1.09, 1.34) | < 0.001 | < 0.02 |
| - Any seasonal vaccination from 2006-2007 to 2008-2009 (Q) | 0 | 0 [0, 0.5] | 1.19 (1.07, 1.32) | < 0.01 | < 0.03 |
| - Using handkerchief when wiping nose (Q) | 0 | 0 [0, 0.33] | 0.99 (0.88, 1.11) | 0.84 | 0.70 |
| - Using (paper) tissue when wiping nose (Q) | 0 | 1 [0.8, 1] | 0.99 (0.87, 1.13) | 0.91 | 0.72 |
| - Using antiviral (paper) tissue when wiping nose (Q) | 0 | 0 [0, 0] | 1.21 (0.62, 2.36) | 0.59 | 0.63 |
| - Always covers mouth while coughing or sneezing (Q) | 1 | 0.5 [0.17, 1] | 1.13 (1.03, 1.23) | < 0.02 | 0.10 |
| - Daily frequency of hand washing  (with soap or hand sanitizer) ≥ 3 (B) (Q) | 1 | 6 [4.33, 8.81] | 1 (0.99, 1.01) | 0.66 | 0.66 |
| - Always washes hands after coughing or sneezing (Q) | 1 | 0 [0, 0] | 1.04 (0.88, 1.23) | 0.63 | 0.66 |
|  |  |  |  |  |  |
|  |  |  |  |  |  |
| **Table S3. Environmental characteristics of the housing** | | | | |  |
| ***General characteristics*** |  |  |  |  |  |
| **Covariate** | **missing values (N)** | **median ([IQR] or N (proportion)** | **GMTR (95% CI)** | **raw P** | **adjusted P** |
| Number of subjects in the household ^(Q)^ | 0 | 3 [2, 4] | 1.01 (0.98, 1.05) | 0.38 | 0.56 |
| Number of children (<15 years) in the household ^(Q)^ | 0 | 0 [0, 2] | 1.01 (0.96, 1.05) | 0.83 | 0.70 |
| Number of other children in the household ^(Q)^ | 0 | 0 [0, 1] | 0.99 (0.94, 1.03) | 0.54 | 0.61 |
| Habitation = house | 0 | 913 (66.3%) | 1.02 (0.94, 1.1) | 0.65 | 0.66 |
| Apartment floor ^(Q)^ | 17 | 0 [0, 1] | 1 (0.98, 1.01) | 0.68 | 0.66 |
| Surface area of habitation (m²) ^(Q)^ | 23 | 100 [80, 135] | 1 (1, 1) | < 0.02 | 0.15 |
| Surface area of habitation per subject (m²) ^(Q)^ | 23 | 36.7 [25, 52.5] | 1 (1, 1) | 0.27 | 0.50 |
| Number of rooms in habitation ^(Q)^ | 34 | 5 [4, 6] | 1.02 (1, 1.04) | 0.11 | 0.41 |
| Number of rooms per subject in habitation ^(Q)^ | 34 | 1.7 [1.3, 2.5] | 1.01 (0.98, 1.05) | 0.41 | 0.56 |
| Ventilation system: |  |  |  |  |  |
| - Air vent | 99 | 642 (50.2%) | 1.01 (0.94, 1.09) | 0.76 | 0.69 |
| - Mechanical ventilation | 48 | 594 (44.7%) | 1 (0.93, 1.08) | 0.92 | 0.72 |
| - Forced-air mechanical ventilation | 84 | 207 (16%) | 1 (0.9, 1.11) | 1.00 | 0.75 |
| - Gas-conduct forced-air mechanical ventilation | 99 | 76 (5.9%) | 1.03 (0.91, 1.16) | 0.65 | 0.66 |
| - Individual | 96 | 155 (12.1%) | 1.03 (0.94, 1.14) | 0.51 | 0.60 |
| Presence of thermostat | 0 | 742 (53.9%) | 1.01 (0.94, 1.09) | 0.78 | 0.70 |
|  |  |  |  |  |  |

| ***General characteristics (continued)*** |  |  |  |  |  |
| --- | --- | --- | --- | --- | --- |
| **Covariate** | **missing values (N)** | **median ([IQR] or N (proportion)** | **GMTR (95% CI)** | **raw P** | **adjusted P** |
| Number of garbage cans used in habitation ^(Q)^ | 22 | 2 [2, 3] | 0.98 (0.96, 1) | 0.11 | 0.43 |
| Presence of cover on garbage cans | 0 | 1205 (87.5%) | 0.98 (0.88, 1.1) | 0.78 | 0.70 |
| Solid garbage placed in plastic bags | 0 | 1260 (91.5%) | 1.05 (0.94, 1.17) | 0.43 | 0.58 |
| Number of times a week garbage pick up ^(Q)^ | 18 | 2 [1, 3] | 1 (0.99, 1.02) | 0.60 | 0.64 |
|  |  |  |  |  |  |
| Prsence of animals inside habitation: |  |  |  |  |  |
| - Any | 0 | 686 (49.8%) | 0.96 (0.89, 1.04) | 0.31 | 0.52 |
| - Presence of dogs | 0 | 366 (26.6%) | 1.02 (0.94, 1.1) | 0.62 | 0.65 |
| - Number of dogs ^(Q)^ | 0 | 0 [0, 1] | 1.01 (0.96, 1.06) | 0.72 | 0.68 |
| - Presence of cats | 0 | 404 (29.3%) | 1.01 (0.93, 1.09) | 0.85 | 0.70 |
| - Number of cats ^(Q)^ | 0 | 0 [0, 1] | 1.02 (0.99, 1.05) | 0.22 | 0.49 |
| - Presence of ferrets | 0 | 4 (0.3%) | 0.79 (0.5, 1.27) | 0.33 | 0.54 |
| - Number of ferrets | 0 | 4 (0.3%) | 0.79 (0.5, 1.27) | 0.33 | 0.54 |
| - Presence of domestic rodents | 0 | 110 (8%) | 0.93 (0.82, 1.05) | 0.27 | 0.50 |
| - Number of domestic rodents ^(Q)^ | 0 | 0 [0, 0] | 0.99 (0.98, 1.01) | 0.57 | 0.62 |
| - Presence of birds | 0 | 55 (4%) | 0.85 (0.73, 0.99) | < 0.05 | 0.27 |
| - Number of birds ^(Q)^ | 0 | 0 [0, 0] | 0.98 (0.97, 0.99) | < 0.001 | < 0.02 |
| Number of bathrooms ^(Q)^ | 0 | 1 [1, 2] | 1.06 (0.98, 1.14) | 0.16 | 0.45 |
| Number of toilets ^(Q)^ | 0 | 2 [2, 2] | 1.07 (1, 1.14) | < 0.05 | 0.27 |
|  |  |  |  |  |  |
|  |  |  |  |  |  |
| Presence of garden/backyard or land surrounding habitation | 0 | 1095 (79.5%) | 1.04 (0.96, 1.13) | 0.30 | 0.52 |
| Surface area garden/backyard, surrounding land around habitation (m²) ^(L)^ | 74 | 5.7 [3.4, 6.8] | 1 (0.99, 1.02) | 0.40 | 0.56 |
|  |  |  |  |  |  |
|  |  |  |  |  |  |
| ***Kitchen*** |  |  |  |  |  |
| **Covariate** | **missing values (N)** | **median ([IQR] or N (proportion)** | **GMTR (95% CI)** | **raw P** | **adjusted P** |
| Surface area of the kitchen (m²) ^(Q)^ | 27 | 12 [9, 15] | 1 (1, 1.01) | 0.40 | 0.56 |
| Presence of a kitchen range hood in the kitchen | 0 | 1026 (74.5%) | 0.98 (0.9, 1.06) | 0.63 | 0.66 |
| Filtration of area | 0 | 736 (53.4%) | 1.03 (0.96, 1.1) | 0.45 | 0.59 |
| Presence of dishwasher | 0 | 944 (68.6%) | 0.96 (0.89, 1.03) | 0.25 | 0.50 |
| Heating: | 0 | 0 [0, 0] | 1 (1, 1) | < 0.0001 | < 0.0001 |
| - Floor heating | 0 | 171 (12.4%) | 1.02 (0.91, 1.14) | 0.71 | 0.68 |
| - Wall heating unit | 0 | 1065 (77.3%) | 0.95 (0.87, 1.03) | 0.22 | 0.49 |
| - Ceiling heating | 0 | 26 (1.9%) | 1.13 (0.96, 1.33) | 0.14 | 0.44 |
| - Central heating | 0 | 667 (48.4%) | 1.05 (0.98, 1.13) | 0.14 | 0.44 |
| - Electric heating | 0 | 399 (29%) | 0.94 (0.87, 1.01) | 0.10 | 0.41 |
| - Fuel heating | 0 | 216 (15.7%) | 1.07 (0.97, 1.18) | 0.15 | 0.45 |
| - Fireplace/chimney heating | 0 | 150 (10.9%) | 0.99 (0.89, 1.1) | 0.85 | 0.70 |
| - Wood heating stove | 0 | 71 (5.2%) | 1 (0.83, 1.19) | 0.96 | 0.73 |
| - Gas heating | 0 | 582 (42.3%) | 1.01 (0.94, 1.09) | 0.72 | 0.68 |
|  |  |  |  |  |  |
| ***Kitchen (continued)*** |  |  |  |  |  |
| **Covariate** | **missing values (N)** | **median ([IQR] or N (proportion)** | **GMTR (95% CI)** | **raw P** | **adjusted P** |
| Flooring: |  |  |  |  |  |
| - Tiles flooring | 0 | 1139 (82.7%) | 1.02 (0.93, 1.11) | 0.74 | 0.69 |
| - Linoleum flooring | 0 | 208 (15.1%) | 0.99 (0.9, 1.09) | 0.83 | 0.70 |
| - Carpeting | 0 | 53 (3.8%) | 0.93 (0.8, 1.09) | 0.38 | 0.56 |
| - Hardwood flooring | 0 | 222 (16.1%) | 0.97 (0.88, 1.06) | 0.47 | 0.59 |
| - Rug / mat flooring | 0 | 42 (3.1%) | 1.03 (0.81, 1.3) | 0.83 | 0.70 |
|  |  |  |  |  |  |
|  |  |  |  |  |  |
| ***Living room*** |  |  |  |  |  |
| **Covariate** | **missing values (N)** | **median ([IQR] or N (proportion)** | **GMTR (95% CI)** | **raw P** | **adjusted P** |
| Surface area ^(Q)^ | 33 | 30 [21.8, 40] | 1 (1, 1) | 0.17 | 0.46 |
| Surface area per subject (m²) ^(Q)^ | 33 | 10 [7.5, 15] | 1 (1, 1.01) | 0.28 | 0.51 |
|  |  |  |  |  |  |
| Temperature ^(Q)^ | 123 | 20 [19, 21] | 1 (0.98, 1.02) | 0.81 | 0.70 |
| Presence of air humidifier | 0 | 66 (4.8%) | 0.92 (0.79, 1.08) | 0.32 | 0.53 |
|  |  |  |  |  |  |
| Heating: |  |  |  |  |  |
| - Floor heating | 0 | 180 (13.1%) | 1.01 (0.91, 1.13) | 0.8 | 0.70 |
| - Wall heating unit | 0 | 1011 (73.4%) | 0.99 (0.91, 1.07) | 0.8 | 0.70 |
| - Ceiling heating | 0 | 22 (1.6%) | 1.06 (0.87, 1.3) | 0.58 | 0.63 |
| - Central heating | 0 | 625 (45.4%) | 1.05 (0.97, 1.12) | 0.21 | 0.48 |
| - Electric heating | 0 | 363 (26.4%) | 0.98 (0.91, 1.07) | 0.7 | 0.67 |
| - Fuel heating | 0 | 215 (15.6%) | 1.07 (0.97, 1.18) | 0.17 | 0.46 |
| - Fireplace/chimney heating | 0 | 203 (14.7%) | 0.94 (0.84, 1.04) | 0.21 | 0.49 |
| - Wood heating stove | 0 | 87 (6.3%) | 1.1 (0.94, 1.28) | 0.22 | 0.49 |
| - Gas heating | 0 | 576 (41.8%) | 1 (0.93, 1.08) | 0.9 | 0.71 |
|  |  |  |  |  |  |
| Flooring: |  |  |  |  |  |
| - Tiles flooring | 0 | 858 (62.3%) | 0.96 (0.89, 1.03) | 0.25 | 0.50 |
| - Linoleum flooring | 0 | 156 (11.3%) | 1.02 (0.91, 1.13) | 0.77 | 0.70 |
| - Carpeting | 0 | 37 (2.7%) | 1.1 (0.85, 1.42) | 0.46 | 0.59 |
| - Hardwood flooring | 0 | 312 (22.7%) | 1.08 (0.99, 1.19) | 0.09 | 0.40 |
| - Rug / mat flooring | 0 | 94 (6.8%) | 1.07 (0.92, 1.23) | 0.38 | 0.56 |
|  |  |  |  |  |  |
|  |  |  |  |  |  |
| ***Bedroom*** |  |  |  |  |  |
| **Covariate** | **missing values (N)** | **median ([IQR] or N (proportion)** | **GMTR (95% CI)** | **raw P** | **adjusted P** |
| Number of subjects in the room ^(Q)^ | 0 | 2 [1, 2] | 0.93 (0.88, 0.99) | < 0.03 | 0.18 |
| Number of children in the room ^(Q)^ | 0 | 0 [0, 0] | 1.08 (0.99, 1.18) | 0.08 | 0.39 |
| Number of other children in the room ^(Q)^ | 0 | 0 [0, 0] | 1.06 (0.92, 1.23) | 0.42 | 0.58 |
| Surface area (m²) ^(Q)^ | 8 | 13 [11, 16] | 1 (1, 1.01) | 0.29 | 0.52 |
| Surface area per subject (m²) ^(Q)^ | 8 | 5 [3, 7.5] | 1 (0.99, 1.01) | 0.76 | 0.69 |
|  |  |  |  |  |  |
| Bedding in natural feathers | 1 | 244 (17.7%) | 0.97 (0.9, 1.05) | 0.48 | 0.59 |
| Double glazing in bedroom | 1 | 1096 (79.7%) | 1 (0.92, 1.09) | 0.97 | 0.73 |
| Temperature ^(Q)^ | 133 | 19 [18, 20] | 1 (0.99, 1.02) | 0.68 | 0.66 |
| Presence of air humidifier | 1 | 85 (6.2%) | 1.07 (0.92, 1.24) | 0.38 | 0.56 |
|  |  |  |  |  |  |

| ***Bedroom (continued)*** |  |  |  |  |  |
| --- | --- | --- | --- | --- | --- |
| **Covariate** | **missing values (N)** | **median ([IQR] or N (proportion)** | **GMTR (95% CI)** | **raw P** | **adjusted P** |
| Windows face: |  |  |  |  |  |
| - Street | 1 | 487 (35.4%) | 0.96 (0.9, 1.03) | 0.29 | 0.51 |
| - Garden | 1 | 734 (53.3%) | 1.03 (0.97, 1.1) | 0.31 | 0.52 |
| - Courtyard | 1 | 216 (15.7%) | 0.99 (0.9, 1.09) | 0.87 | 0.70 |
| - Interior part of habitation | 1 | 8 (0.6%) | 1.01 (0.82, 1.24) | 0.93 | 0.72 |
| - West | 1 | 318 (23.1%) | 1.07 (1, 1.16) | 0.06 | 0.34 |
| - East | 1 | 376 (27.3%) | 1.01 (0.94, 1.08) | 0.81 | 0.70 |
| - South | 1 | 446 (32.4%) | 0.97 (0.9, 1.04) | 0.34 | 0.54 |
| - North | 1 | 291 (21.1%) | 0.96 (0.88, 1.05) | 0.35 | 0.54 |
|  |  |  |  |  |  |
| Heating: |  |  |  |  |  |
| - Floor heating | 1 | 109 (7.9%) | 1.12 (0.98, 1.29) | 0.10 | 0.41 |
| - Wall heating unit | 1 | 1091 (79.3%) | 1.01 (0.93, 1.1) | 0.85 | 0.70 |
| - Ceiling heating | 1 | 18 (1.3%) | 0.87 (0.66, 1.14) | 0.31 | 0.52 |
| - Central heating | 1 | 731 (53.1%) | 1.01 (0.94, 1.08) | 0.76 | 0.69 |
| - Electric heating | 1 | 393 (28.6%) | 0.95 (0.88, 1.03) | 0.23 | 0.49 |
| - Fuel heating | 1 | 206 (15%) | 1.03 (0.94, 1.14) | 0.53 | 0.61 |
| - Gas heating | 1 | 561 (40.8%) | 1.03 (0.96, 1.11) | 0.40 | 0.56 |
|  |  |  |  |  |  |
| Flooring: |  |  |  |  |  |
| - Tiles flooring | 1 | 278 (20.2%) | 0.92 (0.86, 1) | < 0.05 | 0.27 |
| - Linoleum flooring | 1 | 246 (17.9%) | 1.02 (0.93, 1.11) | 0.66 | 0.66 |
| - Carpeting | 1 | 189 (13.7%) | 1.06 (0.96, 1.18) | 0.24 | 0.50 |
| - Hardwood flooring | 1 | 672 (48.8%) | 1.01 (0.94, 1.08) | 0.78 | 0.70 |
| - Rug / mat flooring | 1 | 63 (4.6%) | 0.95 (0.82, 1.09) | 0.45 | 0.59 |
|  |  |  |  |  |  |
|  |  |  |  |  |  |
|  |  |  |  |  |  |
| **Table S4. Attitudes, beliefs and risk perception** | | | | |  |
| ***Beliefs and risk perception*** |  |  |  |  |  |
| **Covariate** | **missing values (N)** | **median ([IQR] or N (proportion)** | **GMTR (95% CI)** | **raw P** | **adjusted P** |
| I feel well informed regarding influenza A | 131 | 710 (70.8%) | 1.02 (0.95, 1.11) | 0.55 | 0.61 |
| Some people were infected by influenza A in France | 162 | 952 (97.9%) | 1.01 (0.88, 1.15) | 0.94 | 0.72 |
| Some people died from influenza A in France | 220 | 867 (94.9%) | 0.98 (0.86, 1.12) | 0.78 | 0.70 |
| Some preventive measures can reduce the infection risk | 126 | 873 (86.6%) | 0.98 (0.89, 1.08) | 0.67 | 0.66 |
| Influenza A can be transmitted by: |  |  |  |  |  |
| - Saliva / droplets | 155 | 969 (99%) | 0.76 (0.54, 1.06) | 0.11 | 0.41 |
| - Nasal mucus | 259 | 755 (86.3%) | 0.9 (0.81, 1.01) | 0.06 | 0.34 |
| - Cough or sneeze | 136 | 991 (99.3%) | 1.32 (0.96, 1.81) | 0.08 | 0.40 |
| - Contact of hands or cheeks | 166 | 915 (94.5%) | 1.02 (0.88, 1.18) | 0.81 | 0.70 |
| - Inert objects | 206 | 811 (87.4%) | 0.99 (0.89, 1.1) | 0.88 | 0.70 |
| - Pork consumption | 308 | 39 (4.7%) | 0.94 (0.8, 1.12) | 0.49 | 0.59 |
| Consequences of influenza A are totally unpredictable | 214 | 645 (70.1%) | 0.99 (0.91, 1.07) | 0.74 | 0.69 |
| Influenza A is most often fatal | 175 | 97 (10.1%) | 0.94 (0.84, 1.06) | 0.34 | 0.54 |
|  |  |  |  |  |  |
| ***Beliefs and risk perception (continued)*** | |  |  |  |  |
| **Covariate** | **missing values (N)** | **median ([IQR] or N (proportion)** | **GMTR (95% CI)** | **raw P** | **adjusted P** |
| Influenza A has a severe impact on bodily functions | 289 | 450 (53.3%) | 0.99 (0.91, 1.07) | 0.73 | 0.69 |
| All infected people have symptoms | 229 | 433 (47.8%) | 0.97 (0.9, 1.04) | 0.35 | 0.54 |
| Prevention against flu highly depends on behaviors | 172 | 799 (83.1%) | 0.9 (0.82, 0.99) | < 0.04 | 0.25 |
| There’s not much we can do to protect ourselves against influenza A | 203 | 293 (31.5%) | 1.01 (0.93, 1.09) | 0.81 | 0.70 |
| We can reduce the infection risk by taking personal measures | 146 | 944 (95.5%) | 1.08 (0.89, 1.3) | 0.46 | 0.59 |
| Influenza A remains a mystery to me | 179 | 411 (43%) | 1.01 (0.93, 1.09) | 0.86 | 0.70 |
| I have a very clear idea about this disease | 194 | 430 (45.7%) | 0.99 (0.92, 1.07) | 0.78 | 0.70 |
| Mechanisms of the disease are not easily understood | 229 | 610 (67.4%) | 1.01 (0.93, 1.1) | 0.80 | 0.70 |
| Efficient measures to avoid being infected: |  |  |  |  |  |
| - Wearing masks in public | 194 | 738 (78.5%) | 0.97 (0.89, 1.06) | 0.49 | 0.59 |
| - Antivirals such as Tamiflu® | 376 | 335 (44.2%) | 1.04 (0.95, 1.13) | 0.42 | 0.57 |
| - Vaccination against seasonal flu | 242 | 305 (34.2%) | 1.08 (0.99, 1.17) | 0.08 | 0.39 |
| - Hand washing | 130 | 985 (98.1%) | 1.1 (0.81, 1.49) | 0.55 | 0.61 |
| - Avoiding public transports | 195 | 634 (67.5%) | 0.98 (0.91, 1.06) | 0.68 | 0.66 |
| - Avoiding public gatherings | 182 | 678 (71.2%) | 1.02 (0.94, 1.1) | 0.62 | 0.66 |
| - Keeping children home from school | 207 | 345 (37.2%) | 1.03 (0.95, 1.11) | 0.49 | 0.59 |
| - Staying home from work | 198 | 266 (28.4%) | 1.06 (0.98, 1.16) | 0.16 | 0.45 |
| - Limiting traffic in infected areas | 179 | 814 (85.2%) | 1 (0.91, 1.1) | 0.99 | 0.74 |
| - Banning street protests | 205 | 479 (51.6%) | 1.04 (0.97, 1.12) | 0.28 | 0.51 |
| - Closing schools and preschools | 200 | 397 (42.5%) | 1.06 (0.99, 1.14) | 0.10 | 0.41 |
| - Placing sick people in quarantine | 159 | 906 (92.9%) | 1.05 (0.92, 1.19) | 0.48 | 0.59 |
| There's a high probability (≥50%) of being infected: |  |  |  |  |  |
| - If no preventive measure is taken ^(Q)^ | 152 | 40 [10, 50] | 1 (1, 1) | 0.31 | 0.52 |
| - If preventive measures are taken ^(Q)^ | 147 | 15 [5, 30] | 1 (1, 1) | 0.85 | 0.70 |
| Influenza A is a severe disease ^(Q)^ | 142 | 5 [3.8, 7] | 1 (0.99, 1.02) | 0.87 | 0.70 |
| I am confident in authorities to mitigate an epidemic | 167 | 602 (62.3%) | 1.01 (0.93, 1.09) | 0.88 | 0.70 |
| I am confident in authorities to inform me about influenza A | 152 | 643 (65.5%) | 1.02 (0.95, 1.11) | 0.55 | 0.61 |
| Authorities hide important information about influenza A | 273 | 397 (46.1%) | 1 (0.93, 1.08) | 0.97 | 0.74 |
|  |  |  |  |  |  |
|  |  |  |  |  |  |

| ***Attitudes*** |  |  |  |  |  |
| --- | --- | --- | --- | --- | --- |
| **Covariate** | **missing values (N)** | **median ([IQR] or N (proportion)** | **GMTR (95% CI)** | **raw P** | **adjusted P** |
| I have already taken preventive measures against influenza | 171 | 911 (94.6%) | 1.12 (0.94, 1.33) | 0.20 | 0.48 |
| In case of epidemic, I would intend to: |  |  |  |  |  |
| - Wear masks in public | 125 | 27 (2.7%) | 0.91 (0.69, 1.21) | 0.53 | 0.61 |
| - Take antivirals such as Tamiflu® | 125 | 6 (0.6%) | 1.24 (0.8, 1.94) | 0.34 | 0.54 |
| - Get vaccinated against seasonal flu | 127 | 261 (25.9%) | 1.19 (1.09, 1.3) | < 0.001 | < 0.01 |
| - Often wash hands | 127 | 882 (87.6%) | 1.07 (0.96, 1.18) | 0.23 | 0.50 |
| - Avoid public transports | 165 | 259 (26.7%) | 1.01 (0.93, 1.09) | 0.84 | 0.70 |
| - Avoid public gatherings | 160 | 233 (23.9%) | 1.07 (0.98, 1.16) | 0.12 | 0.43 |
| - Keep children home from school | 283 | 110 (12.9%) | 0.97 (0.88, 1.07) | 0.55 | 0.61 |
| - Stay home from work | 215 | 26 (2.8%) | 1.1 (0.85, 1.43) | 0.46 | 0.59 |
|  |  |  |  |  |  |
|  |  |  |  |  |  |
| **Table S5. Nature and daily duration of meetings (minutes)** | | | | | |
| **Covariate** | **missing values (N)** | **median ([IQR] or N (proportion)** | **GMTR (95% CI)** | **raw P** | **adjusted P** |
| Duration of meetings: |  |  |  |  |  |
| - All ^(L)^ | 17 | 6.9 [6.2, 7.4] | 1.04 (1, 1.08) | < 0.05 | 0.28 |
| - At home ^(L)^ | 17 | 6.1 [5.5, 6.6] | 1 (0.98, 1.01) | 0.80 | 0.70 |
| - At school ^(L)^ | 17 | -6.9 [-6.9, 3.9] | 1.01 (1.01, 1.02) | < 0.0001 | < 0.01 |
| - At work ^(L)^ | 17 | -6.9 [-6.9, 5.5] | 0.99 (0.99, 1) | < 0.02 | 0.15 |
| - In transport ^(L)^ | 17 | -6.9 [-6.9, -6.9] | 1 (1, 1.01) | 0.30 | 0.52 |
|  |  |  |  |  |  |
| With subjects aged: |  |  |  |  |  |
| - 0-10 years ^(L)^ | 17 | 2.1 [-6.9, 5.4] | 1 (0.99, 1.01) | 0.90 | 0.71 |
| - 10-20 years ^(L)^ | 17 | 3.2 [-6.9, 5.5] | 1.01 (1, 1.01) | 0.05 | 0.32 |
| - 20-30 years ^(L)^ | 17 | 2.6 [-6.9, 4.9] | 1 (0.99, 1) | 0.43 | 0.58 |
| - 30-60 years ^(L)^ | 17 | 6 [5.2, 6.5] | 0.99 (0.97, 1) | 0.15 | 0.45 |
| - > 60 years ^(L)^ | 17 | 5 [3.4, 5.7] | 1 (0.99, 1.01) | 0.66 | 0.66 |
|  |  |  |  |  |  |
|  |  |  |  |  |  |
| **Table S6. Geographic area** |  |  |  |  |  |
| ***Activity near habtation*** |  |  |  |  |  |
| **Covariate** | **missing values (N)** | **median ([IQR] or N (proportion)** | **GMTR (95% CI)** | **raw P** | **adjusted P** |
| Agricultural land | 0 | 718 (52.1%) | 0.94 (0.88, 1.01) | 0.09 | 0.40 |
|  |  |  |  |  |  |
| Presence of farming: |  |  |  |  |  |
| - Any | 0 | 71 (5.2%) | 0.99 (0.85, 1.15) | 0.85 | 0.70 |
| - Poultry | 0 | 56 (4.1%) | 1.02 (0.85, 1.22) | 0.86 | 0.70 |
| - Pork | 0 | 20 (1.5%) | 0.91 (0.74, 1.12) | 0.39 | 0.56 |
| Factory | 0 | 317 (23%) | 0.94 (0.86, 1.03) | 0.16 | 0.45 |
|  |  |  |  |  |  |

| ***Demography*** |  |  |  |  |  |
| --- | --- | --- | --- | --- | --- |
| **Covariate** | **missing values (N)** | **median ([IQR] or N (proportion)** | **GMTR (95% CI)** | **raw P** | **adjusted P** |
| Proportion of inhabitants aged: |  |  |  |  |  |
| - < 15 years ^(Q)^ | 0 | 0.18 [0.16, 0.2] | 0.47 (0.2, 1.11) | 0.09 | 0.40 |
| - 15-30 years ^(Q)^ | 0 | 0.17 [0.15, 0.21] | 1.03 (0.61, 1.74) | 0.90 | 0.71 |
| - 30-45 years ^(Q)^ | 0 | 0.2 [0.19, 0.23] | 0.52 (0.17, 1.64) | 0.26 | 0.50 |
| - 45-60 years ^(Q)^ | 0 | 0.2 [0.18, 0.23] | 1.87 (0.57, 6.21) | 0.30 | 0.52 |
| - 60-75 years ^(Q)^ | 0 | 0.14 [0.11, 0.16] | 1.04 (0.38, 2.83) | 0.94 | 0.72 |
| - >75 years ^(Q)^ | 0 | 0.08 [0.06, 0.11] | 1.84 (0.73, 4.64) | 0.20 | 0.48 |
| Proportion of inhabitants living at the same place 5 years ago ^(Q)^ | 0 | 0.67 [0.61, 0.73] | 0.76 (0.53, 1.1) | 0.14 | 0.44 |
| Proportion of foreigners ^(Q)^ | 0 | 0.03 [0.01, 0.07] | 1.59 (0.86, 2.96) | 0.14 | 0.44 |
| Proportion of migrants ^(Q)^ | 0 | 0.06 [0.03, 0.11] | 1.35 (0.81, 2.23) | 0.24 | 0.50 |
| Proportion of unemployment among 15-65 years ^(Q)^ | 0 | 0.08 [0.06, 0.1] | 0.95 (0.3, 3.07) | 0.94 | 0.72 |
| Socio-professional groups (proportion): |  |  |  |  |  |
| - Farmer, primary sector ^(Q)^ | 0 | 0 [0, 0.01] | 0.7 (0.26, 1.89) | 0.48 | 0.59 |
| - Craftsman, shopkeeper, chief executive officer ^(Q)^ | 0 | 0.03 [0.02, 0.05] | 2 (0.35, 11.3) | 0.43 | 0.58 |
| - Executive, intellectual profession ^(Q)^ | 0 | 0.08 [0.05, 0.12] | 1.4 (0.89, 2.21) | 0.14 | 0.44 |
| - Middle class ^(Q)^ | 0 | 0.16 [0.14, 0.19] | 0.35 (0.16, 0.77) | < 0.01 | 0.10 |
| - Employee ^(Q)^ | 0 | 0.18 [0.15, 0.21] | 0.77 (0.34, 1.71) | 0.52 | 0.60 |
| - Working class ^(Q)^ | 0 | 0.14 [0.1, 0.19] | 1.07 (0.6, 1.91) | 0.82 | 0.70 |
| Proportion of inhabitants working in same commune ^(Q)^ | 0 | 0.19 [0.12, 0.38] | 1.32 (1.04, 1.68) | < 0.03 | 0.18 |
| Proportion of inhabitants working in same department but different commune ^(Q)^ | 0 | 0.26 [0.14, 0.46] | 0.88 (0.72, 1.07) | 0.21 | 0.48 |
| Proportion of workers using public transports to go to work ^(Q)^ | 0 | 0.05 [0.02, 0.1] | 1.35 (1.00, 1.82) | < 0.05 | 0.28 |
| Proportion of households with: |  |  |  |  |  |
| - persons living alone ^(Q)^ | 0 | 0.28 [0.22, 0.41] | 1.13 (0.86, 1.49) | 0.37 | 0.56 |
| - families with children ^(Q)^ | 0 | 0.3 [0.22, 0.37] | 0.76 (0.54, 1.08) | 0.12 | 0.44 |
| - single-parent families ^(Q)^ | 0 | 0.08 [0.06, 0.11] | 0.59 (0.26, 1.31) | 0.19 | 0.48 |
| - couples with no children ^(Q)^ | 0 | 0.28 [0.22, 0.33] | 1.26 (0.74, 2.13) | 0.4 | 0.56 |
| - families with 3 children ^(Q)^ | 0 | 0.04 [0.03, 0.06] | 0.32 (0.08, 1.37) | 0.13 | 0.44 |
| - families with more than 3 children ^(Q)^ | 0 | 0.01 [0.01, 0.02] | 0.31 (0.13, 0.76) | < 0.02 | 0.10 |
| Proportion of inhabitants > 15 years: |  |  |  |  |  |
| - Not going to school ^(Q)^ | 0 | 0.9 [0.87, 0.91] | 1.14 (0.64, 2.03) | 0.66 | 0.66 |
| - Without a diploma ^(Q)^ | 0 | 0.56 [0.46, 0.64] | 0.99 (0.75, 1.31) | 0.95 | 0.73 |
| - Not attending higher education ^(Q)^ | 0 | 0.08 [0.05, 0.13] | 1.32 (0.86, 2.01) | 0.20 | 0.48 |
| Proportion of houses (vs. other habitations) ^(Q)^ | 0 | 0.67 [0.21, 0.91] | 0.94 (0.86, 1.04) | 0.25 | 0.50 |

| ***Demography*** |  |  |  |  |  |
| --- | --- | --- | --- | --- | --- |
| **Covariate** | **missing values (N)** | **median ([IQR] or N (proportion)** | **GMTR (95% CI)** | **raw P** | **adjusted P** |
| Proportion of habitations: |  |  |  |  |  |
| - With surface > 40 m^2^ ^(Q)^ | 0 | 0.05 [0.02, 0.12] | 1.29 (0.95, 1.74) | 0.10 | 0.41 |
| - With surface > 100 m^2^ ^(Q)^ | 0 | 0.28 [0.14, 0.45] | 0.86 (0.71, 1.05) | 0.14 | 0.44 |
| - Owned by inhabitants ^(Q)^ | 0 | 0.64 [0.45, 0.79] | 0.9 (0.77, 1.05) | 0.19 | 0.48 |
| - Social housing ^(Q)^ | 0 | 0.07 [0.01, 0.19] | 1.04 (0.87, 1.25) | 0.64 | 0.66 |
| - With bathroom ^(Q)^ | 0 | 0.97 [0.96, 0.98] | 0.65 (0.16, 2.66) | 0.55 | 0.61 |
| - With collective heating system ^(Q)^ | 0 | 0.06 [0.02, 0.25] | 1.06 (0.92, 1.22) | 0.43 | 0.58 |
| - With private electric heating ^(Q)^ | 0 | 0.22 [0.12, 0.33] | 1.18 (0.93, 1.49) | 0.18 | 0.47 |
| Proportion with car ownership ^(Q)^ | 0 | 0.86 [0.74, 0.93] | 0.82 (0.63, 1.06) | 0.13 | 0.44 |
| Mean annual income (k€) ^(Q)^ | 0 | 22.1 [19.5, 26.5] | 1.00 (1.00, 1.01) | 0.30 | 0.52 |
