# Supplementary material for: Factors Associated with Post-Seasonal Serological Titer and Risk Factors for Infection with the Pandemic A/H1N1 Virus in the French General Population
Source: PLoS One. 2013 Apr 16;8(4):e60127. doi: 10.1371/journal.pone.0060127 (PMC3629047; doi:10.1371/journal.pone.0060127)
Supplement: File S2 — Tables S7 and S8. Multivariable models for the case-control analysis of risk factors for probable infection in CoPanFlu-France unvaccinated subjects: sensitivity analysis. (DOC) [file pone.0060127.s002.doc]

**Factors associated with post-seasonal serological titer and risk factors for infection with the pandemic A/H1N1 virus in the French general population**

Supplementary tables S7 and S8: Multivariable models for the case-control analysis of risk factors for probable infection in CoPanFlu-France unvaccinated subjects: sensitivity analysis

All covariates are binary except ^(Q)^ quantitative covariates and ^(L)^ log-transformed quantitative covariates.

**Table S7. Sensitivity analysis (1): subjects without pandemic vaccination, 171 cases with HI titer ≥ 1/80, 512 controls with HI titer < 1/40**

| **Covariate** | **OR (95% CI)** | **P** |
| --- | --- | --- |
| Age at inclusion (per 10 years) ^(Q)^ | 0.87 (0.77, 0.98) | < 0.03 |
| Seasonal vaccine recipient (any year from 2006 to 2008) | 2.29 (1.43, 3.67) | < 0.001 |
| Chronic obstructive pulmonary disease | 2.96 (1.41, 6.22) | < 0.01 |
| Asthma | 2.14 (1.12, 4.11) | < 0.03 |
| Duration of meetings at school ^(L)^ | 1 .12 (1.04, 1.21) | < 0.01 |
| Air humidifier in the living room | 0.41 (0.17, 0.96) | < 0.04 |
| *Pairwise odds ratios between cases living in the same household* | *5.13 (2.39, 11.0)* | *< 0.001* |

**Table S8. Sensitivity analysis (2): subjects without pandemic vaccination, 30 cases with HI titer ≥ 1/80 and history of ILI, 605 controls with HI titer < 1/40 and no history of ILI**

| **Covariate** | **OR (95% CI)** | **P** |
| --- | --- | --- |
| Age at inclusion (per 10 years) ^(Q)^ | 0.69 (0.56, 0.86) | < 0.01 |
| Chronic obstructive pulmonary disease | 6.52 (1.99, 21.3) | < 0.01 |
| Asthma | 3.43 (1.48, 7.94) | < 0.01 |
| *Pairwise odds ratios between cases living in the same household* | *5.39 (2.27, 12.8)* | *< 0.001* |
